# Electrochemical Behaviors of Methylene Blue on DNA Modified Electrode and Its Application to the Detection of PCR Product from NOS Sequence

**DOI:** 10.3390/s8095649

**Published:** 2008-09-15

**Authors:** Ling Zhu, Ruijun Zhao, Kegang Wang, Haibo Xiang, Zhimei Shang, Wei Sun

**Affiliations:** 1 Zibo Entry-Exit Inspection and Quarantine Bureau of P.R. China, Zibo 255031, Shandong, P.R. China; 2 College of Chemistry and Molecular Engineering, Qingdao University of Science and Technology, Qingdao 266042, Shandong, P.R. China

**Keywords:** Electrochemical DNA biosensor, methylene blue, polymerase chain reaction, NOS gene, hybridization

## Abstract

An electrochemical DNA biosensor for the detection of NOS gene sequences from genetically modified organisms (GMOs) is presented in this paper. Single-stranded DNA (ssDNA) was covalently attached through the carboxylate ester formed by the 3′-hydroxy end of the DNA with the carboxyl of a mercaptoacetic acid self-assembled monolayer-modified gold electrode using *N*-hydroxysuccinimide (NHS) and *N*-(3-dimethylaminopropyl)-*N′*-ethylcarbodiimide hydrochloride (EDC) as linkers. The electrochemical behavior of methylene blue (MB) on the ssDNA and dsDNA modified gold electrode were carefully studied. Compared with ssDNA/Au electrode, an increase of redox peak current of MB on dsDNA/Au electrode was found, which could be further used for monitoring the recognition of DNA hybridization. Based on this result, the polymerase chain reaction (PCR) product of the common inserts NOS terminator from real GMOs samples was detected successfully.

## 1. Introduction

The detection of specific DNA sequences is important in the fields of biochemistry and life science. Among the methods for DNA detection, an electrochemical DNA hybridization biosensor is suitable for the study of specific sequence DNA with low detection limits and high sensitivity [1-4]. It is based on the immobilization of an oligonucleotide on the electrode surface and detection of the hybridization procedure with different electrochemical techniques. Many methods have been reported for the immobilization of the ssDNA probe on an electrode surface, including direct adsorption [5-6], avidin-biotin system [7], self-assembled methods [8-9], covalent bonding [10-12] and nanoparticles [13-16] etc. Among them a self-assembled monolayer (SAM) of alkanethiols on a gold electrode has been proven to be an effective way for the DNA immobilization with the advantages of simplicity, high level of orientation and versatility [17]. Through the strong Au-S linkage an ordered film can be formed on the surface of gold electrode. Millan *et al.* [18] described a sequence-selective biosensor on self-assembled monolayer (SAM) glassy carbon electrode for DNA with Co (bpy)_3_^3+^ as indicator. Sun *et al.* [19] immobilized ssDNA on an aminoethanethiol monolayer-modified gold electrode. Ju *et al.* [20] applied 2-mercaptoethylamine self-assembled gold electrode in the DNA biosensor for the immobilization of yeast DNA.

In this paper the electrochemical behaviour of methylene blue (MB) on a mercaptoacetic acid self-assembled monolayer DNA modified gold electrode was investigated. MB is a commonly used electrochemical indicator [21-23] for DNA biosensors and it was selected for the experiments to detect DNA hybridization, which showed different electrochemical responses on ssDNA or dsDNA modified electrodes. The DNA biosensor was further used for the detection of the polymerase chain reaction (PCR) product of a nopaline synthase (NOS) gene sequence, which is commonly used as terminator inserted into genetically modified organisms (GMOs). The detection of NOS segments in real samples will prove them to be the product of transgenic plants. Since the PCR method is an effective technique for the target DNA amplification, the combination of PCR amplification with electrochemical detection provides a sensitive method for specific sequence detection. The schedule for the preparation of DNA modified electrode was illustrated as follows.

## 2. Experimental Section

### 2.1 Apparatus

All the cyclic voltammetric measurements were performed on a LK 98A electrochemical analyzer (Tianjin Lanlike Chemical and Electron High Technology Co. LTD, P.R. China) with a three-electrode system consisted of a bare gold or DNA modified gold electrode as working electrode, a saturated calomel electrode (SCE) as reference electrode and a platinum wire as auxiliary electrode. The PCR amplification was performed on an Eppendorf Mastercycler Gradient PCR system (Eppendorf, Germany). A pHS-25 acidimeter (Shanghai Leici Instrument Factory, P.R. China) was used to control the pH of buffer solutions.

### 2.2 Reagents

Methylene blue (MB, Shanghai Reagent Company), mercaptoacetic acid (MAA, Tianjin Yuanhang Reagent Company), *N*-hydroxysuccinimide (NHS, Shanghai Reagent Company), *N*-(3-dimethylamino-propyl)-*N′*-ethylcarbodiimide hydrochloride (EDC, Sigma) and native fish sperm dsDNA (Beijing Jingke Reagent Company) were used as received and fish sperm ssDNA was produced by heating the native dsDNA solution in a 100°C water bath for about 10 min and then cooling in an ice bath.

The buffers were prepared as follows: phosphate buffer solution (PBS, 20.0 mmol/L KH_2_PO_4_+ NaOH, pH 7.0), 2×SSC hybridization buffer (300.0 mmol/L NaCl + 30.0 mmol/L sodium citrate tribasic dihydrate, pH 7.0), Tris-EDTA buffer solution (TE, 10.0 mmol/L Tris-HCl+1.0 mmol/L EDTA), 0.2 mol/L Britton-Robinson (B-R) buffer solution (pH 6.0). All the other reagents were of analytical reagents and doubly distilled water was used throughout.

### 2.3 Polymerase chain reaction procedure

The DNA sample for PCR amplification was extracted from transgenic soybean with a plant DNA Mini Preparation Kit purchased from Shanghai Academy of Agricultural Sciences. The PCR reaction was performed on an Eppendorf Mastercycler Gradient PCR system using oligonucleotide primers for NOS with the following sequences:

NOS Primer 1: 5′-GAATCCTGTTGCCGGTCTTG-3′;
NOS Primer 2: 5′-TTATCCTAGTTTGCGCGCTA-3′.

NOS, a common insert in GMOs, is the terminator of the nopaline synthase gene. Amplification of NOS DNA fragments was carried out in a final volume of 25 µL in 0.2 mL tube containing 200.0 nmol/L each primer of NOS Primer 1 and NOS Primer 2, 10×reaction buffer B (Promega, Wisconsin USA), 2.0 mmol/L MgCl_2_, 200.0 nmol/L each of dATP, dCTP, dGTP and dTTP; 1.5 units of Taq DNA polymerase (Promega, Wisconsin USA), 1.0 µL DNA template purified from samples.

During the PCR procedure, DNA was initial denatured at 94°C for 30 s. PCR condition was optimized as follows: 35 cycles of amplification (94°C for 30 s, 56°C for 30 s, 72°C for 30 s) and final extension at 72 °C for 5 min. 6 µL each of the PCR products were analyzed by electrophoresis separation (5 V/cm, 40 min) on a 2% agarose gel containing 0.5 µg/mL of ethidium bromide in 1×TAE buffer (40.0 mmol/L Tris, 1.0 mmol/L EDTA, 40.0 mmol/L acetate, pH 8.0), and visualized with UV transilluminator. The PCR products of NOS gene were 195 bp fragments and kept at 4°C before use.

### 2.4 Preparation of self-assembled gold electrode

The surface of gold electrode was treated step by step with the following procedure. The gold electrode was first heated in a Piraña solution (30% H_2_O_2_ and concentrated H_2_SO_4_ in 3:7) for about 5 min and then polished with 0.05 µm albumin/water slurry on polishing cloth to get a mirror-like surface. After dipping in a HNO_3_ solution (1:1) for 30 min, the electrode was sonificated in ethanol and water for 2 min each, respectively. The extent of electrode surface treatment was evaluated by cyclic voltammetric measurement in 1.0 mmol/L K_3_Fe(CN)_6_ solution. The freshly pretreated gold electrode was dipped into a 10.0 mmol/L mercaptoacetic acid (MAA) solution for about 24 h to get the MAA self-assembled gold electrode. After that, the electrode was rinsed with water to remove the physically adsorbed MAA and further stored in distilled water. The MAA monolayer modified electrode was then denoted as MAA/Au electrode.

### 2.5 Immobilization of ssDNA on MAA/Au electrode

The MAA/Au electrode was immersed in pH 7.0 PBS containing 5.0 mmol/L EDC and 8.0 mmol/L NHS for 30 min to get a Au/MAA/Linker electrode, then dipped in ssDNA solution (pH 7.0, PBS) for 12 h at 25°C. At this step the covalent bonding between carboxyl groups and 3′-hydroxy end of DNA was occurred to form a carboxylate ester and ssDNA was immobilized on the surface of gold electrode. After washed thoroughly with water to remove the non-specifically absorbed ssDNA, an ssDNA-modified electrode (denoted as ssDNA/MAA/Au) was obtained.

### 2.6 Hybridization on the electrode surface

The ssDNA/MAA/Au electrode was immersed into target ssDNA in 2×SSC buffer solution for 1 h at 60 °C and then cooled gradually to the room temperature. After hybridization, the electrode was rinsed by water and a dsDNA/MAA/Au electrode was got, which was stored in a pH 8.0 TE buffer solution at 4°C before use.

### 2.7 Electrochemical measurement

Cyclic voltammetry was used in the electrochemical measurements with the scan rate as 100 mV/s. By immersing the DNA modified electrode in 1.5×10^−4^ mol/L MB at pH 6.0 Britton-Robinson (B-R) buffer solutions for 5 min and scanned in the potential range from -0.40 V to 0.10 V, the redox signal of the accumulated MB was recorded. All the results were the average value of three parallel measurements.

## 3. Results and Discussion

### 3.1 Electrochemical studies of MB with ssDNA and dsDNA in solution

**Figure 1.**
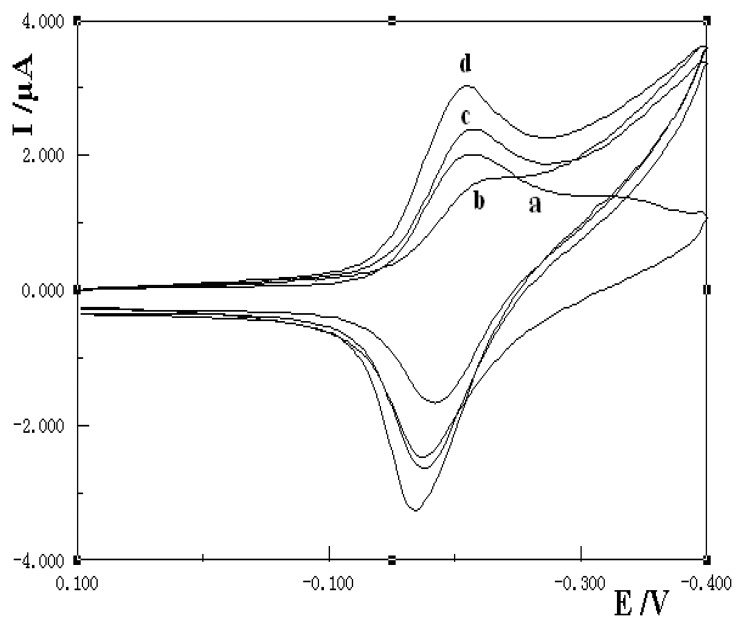
Cyclic voltammograms of 1.5×10^−4^ mol/L MB on different kinds of electrodes; a) bare Au; b) MAA/Au; c) ssDNA/MAA/Au; d) dsDNA/MAA/Au in 0.2 mol/L pH 6.0 B-R buffer, scan rate: 100 mV/s.

The interaction of MB with ssDNA and dsDNA in solution was first studied by cyclic voltammetry. The electrochemical behavior of MB had been reported as a two electrons and one proton electrode process [22] and in the selected conditions MB showed a pair of symmetrical redox peaks (curve a of Figure 1). After the addition of 100.0 mg/L ssDNA and dsDNA into the MB solution, both of the redox peaks current decreased with different value, which indicated that ssDNA and dsDNA could interact with MB with different binding mode. The electrochemical data of MB with the addition of ssDNA and dsDNA were summarized in Table 1.

Generally three kinds of binding modes including electrostatic, groove and intercalative binding take place between small molecules and DNA. The binding properties depend on the both the nature of small molecules and the experimental conditions, such as the ionic strength, the concentration of small molecules and DNA, and the buffer pH *etc.* From Table 1 it can be seen that the redox peak currents of MB decreased more in dsDNA solution than in ssDNA solution, which indicated that MB had higher affinity towards dsDNA than ssDNA. According to [24], MB can interact with ssDNA by electrostatic binding, while the effect of electrostatic and intercalation binding coexist in a dsDNA solution, so under the selected conditions a much greater decrease of redox peaks was observed upon the addition of dsDNA, indicating that dsDNA had stronger binding ability with MB than that of ssDNA and MB. This can be used to distinguish between ssDNA and dsDNA.

### 3.2 Cyclic voltammogram of MB on different modified electrodes

Figure 1 shows the electrochemical behavior of 1.5×10‐^4^ mol/L MB on different modified electrodes in pH 6.0 Britton-Robinson (B-R) buffer solution with the scan rate of 100 mV/s. Curve a is the cyclic voltammogram of MB on a bare gold electrode. A pair of symmetrical redox peaks appeared, due to the electrochemical reduction of MB to leucomethylene blue. Curve b is that of MB on MAA self-assembled monolayer modified gold electrode; in this case the redox peak currents apparently decreased with a negative peak potential shift. The redox peak potentials obtained were Epa= – 0.182 V and Epc= – 0.242 V with ΔEp=56 mV, which was bigger than that of MB. The result indicated that it was difficult for MB to participate in the redox reaction on the surface of MAA/Au electrode, which may be due to the formation of MAA self-assembled monolayer on the gold electrode surface which hindered the diffusion of MB molecule to the electrode surface and resulted in the decrease of peak current. Curve c is the cyclic voltammogram of MB on a ssDNA/MAA/Au electrode.

Compared with those of curve a and curve b, the redox peak currents increased, which indicated that ssDNA had been successfully immobilized on the surface of MAA/Au electrode. The redox peak potentials were negatively moved with the E_pa_= − 0.176 V and E_pc_= −0.216 V, which is characteristic of an electrostatic attraction. The result was in accordance with [25], which indicated that more MB was accumulated on the electrode surface and resulted in the increase of the redox peak currents. Curve d was cyclic voltammogram of MB on dsDNA/MAA/Au electrode. The peak potentials moved to − 0.168 V and −0.210 V, respectively. The peak current increased greatly with a positive movement of redox peak potentials, which is typical of intercalation of MB with dsDNA. The MB molecules had intercalated into the double helix structure of immobilized dsDNA. More MB was concentrated on the dsDNA/MAA/Au surface and this resulted in a great redox peak current increase. The results were not consistent with same previous reports based on the interaction of MB with guanine residues of DNA, which showed higher electrochemical responses on ssDNA than the dsDNA modified electrode [22, 26]. In reference [25] phenomena similar to those reported in this paper were noted. This may be due to the higher concentration of MB used in this experiment, which induced the binding model from the surface electrostatic attraction on ssDNA molecules to the intercalation on the dsDNA molecules. The difference of the electrochemical response of MB on ssDNA/MAA/Au and dsDNA/MAA/Au electrode indicated that MB was a good indicator to distinguish the ssDNA or dsDNA on the surface of SAM/Au electrode.

### 3.3 Electrochemical behavior of MB on ssDNA or dsDNA modified electrode

**Figure 2.**
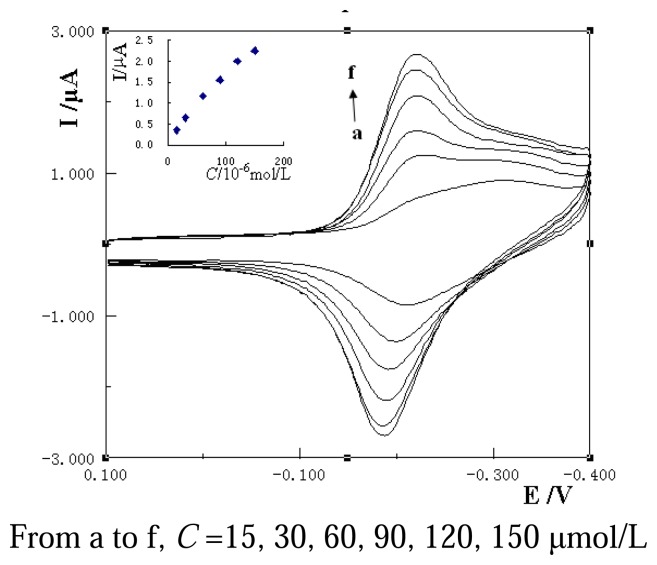
Influence of MB concentrations on peak current of ssDNA/MAA/Au electrode.

Cyclic voltammograms of different MB concentrations on ssDNA/MAA/Au or dsDNA/MAA/Au electrode were recorded. Figure 2 shows the results of different amounts of MB on the ssDNA/MAA/Au electrode. With the increase of the concentration of MB, the peak currents increased and reached a maximum value. The plot of the peak current with the concentration of MB was shown in the inset of Figure 2, which exhibited an expected shape of Langumuir adsorption [27]. According to the Langumuir adsorption thermodynamic equation:

$$\frac{C}{I_{p}}=\frac{1}{KI_{p,\max}}+\frac{C}{I_{p,\max}}$$

where *I_p_* represents the anodic or cathodic peak current, *I_p,max_* the maximum peak current, *C* the concentration of MB and *K* the adsorption constant of MB on the DNA modified electrode. From the slope and the intercept of the plot of *C*/*I_p_* vs. *C*, the values of *I_p,max_* and *K* can be obtained.

**Figure 3.**
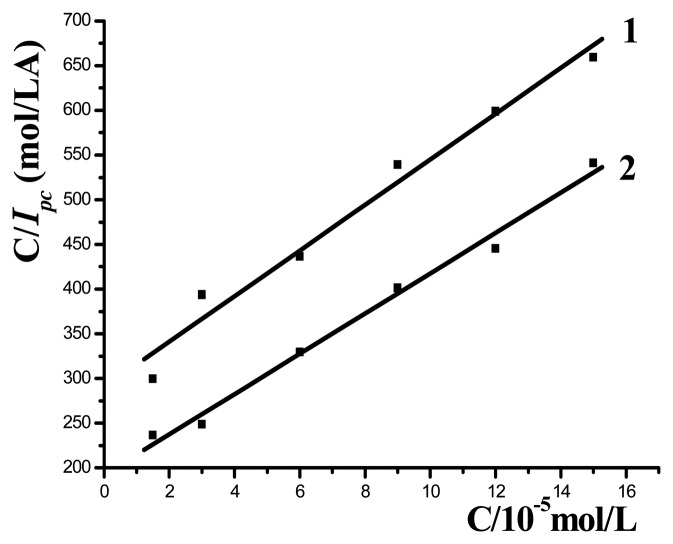
Plot of *C*/*I_pc_* vs. *C* at ssDNA/MAA/Au (1) and dsDNA/MAA/Au electrodes (2).

Figure 3 shows the plots of C/*I_pc_* vs. *C* at ssDNA/MAA/Au (curve 1) and dsDNA/MAA/Au (curve 2) electrode, respectively, with two lines got. From the intercept and slope the adsorption constants (*K*) of MB on ssDNA/MAA/Au and dsDNA/MAA/Au electrode were calculated as 8.5×10^3^ L/mol and 1.13×10^4^ L/mol, respectively. The increase of adsorption constants indicated the MB had stronger affinity with dsDNA than ssDNA and the complex of MB with immobilized dsDNA was more stable than that with ssDNA.

**Figure 4.**
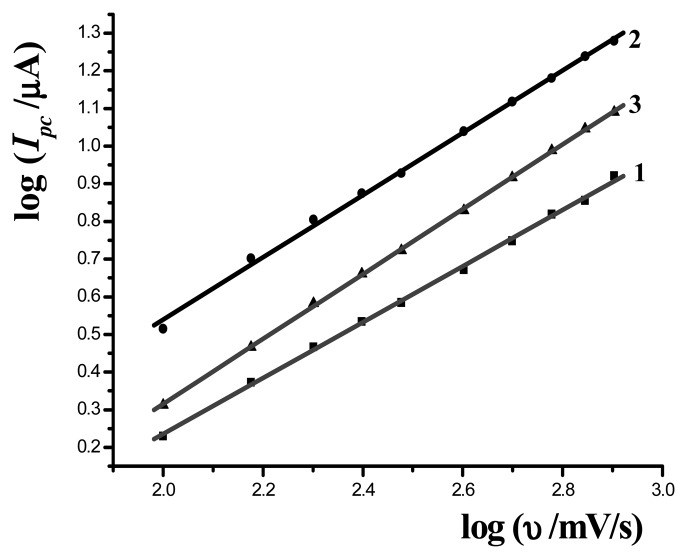
Plots of cathodic peak current vs. log υ on bare Au electrode (1), ssDNA/MAA/Au (2) and dsDNA/MAA/Au electrode (3).

The influence of scan rate (υ) on the redox peak current of MB at different modified electrodes was also investigated in the range from 50 to 800 mV/s. With the increase of scan rate, both of the reductive and oxidative peak currents increased. Figure 4 shows the plots of log*I_pc_* vs. logυ at bare gold electrode (curve 1), ssDNA/MAA/Au (curve 2) and dsDNA/MAA/Au electrode (curve 3), respectively. Three lines were got with the slope as 0.74, 0.82 and 0.86, respectively. The results were between 0.5 and 1, which was the ideal value of diffusion-controlled process and surface-controlled process, respectively. So the electrochemical process of MB on the electrodes was the combination of diffusion-controlled and surface-controlled. The values also indicated that the slopes were increased gradually on bare, ssDNA and dsDNA modified electrode with the result more close to 1, so the contribution of surface-controlled process on dsDNA modified electrode was more than that on ssDNA modified electrode, which was due to that more MB was bound to dsDNA on the surface of dsDNA/MAA/Au electrode.

### 3.4 Calibration curve

**Figure 5.**
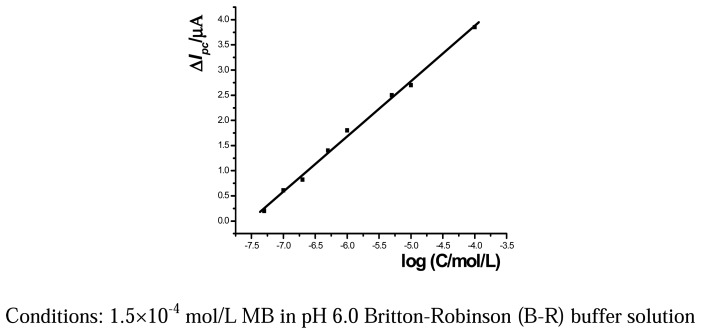
Calibration curve for the ssDNA sequence detection.

The established electrochemical biosensor was further applied to the detection of ssDNA sequences. After hybridization with different amounts of ssDNA the cathodic peak current was increased gradually in the concentration range from 5.0×10^-8^ to 1.0×10^-4^ mol/L with the results shown in Figure 5. The linear regression equation was calculated as Δ*I_pc_* (µA)=1.10 logC (mol/L) +8.25 (n=8, γ =0.9986) and the detection limit as 3.60×10^-8^ mol/L (3σ). The relative standard deviation (RSD) of 11 parallel determinations of 1.0×10^-7^ mol/L ssDNA was estimated as 3.89%.

### 3.5 Application to the detection of PCR product from the NOS gene

The proposed electrochemical DNA biosensor was further applied to the directly detection of PCR product of NOS gene. The NOS gene template was extracted from the transgenic modified soybean with the DNA Mini Preparation Kit. The PCR product of NOS gene was diluted with 5.0 mmol/L Tris-HCl buffer and denatured by heating it in boiling water for 5 min and then freezing in an ice bath for 2 min. The primer 1 of NOS gene was immobilized on the MAA/Au with the established method to obtain a NOS ssDNA/MAA/Au electrode.

**Figure 6.**
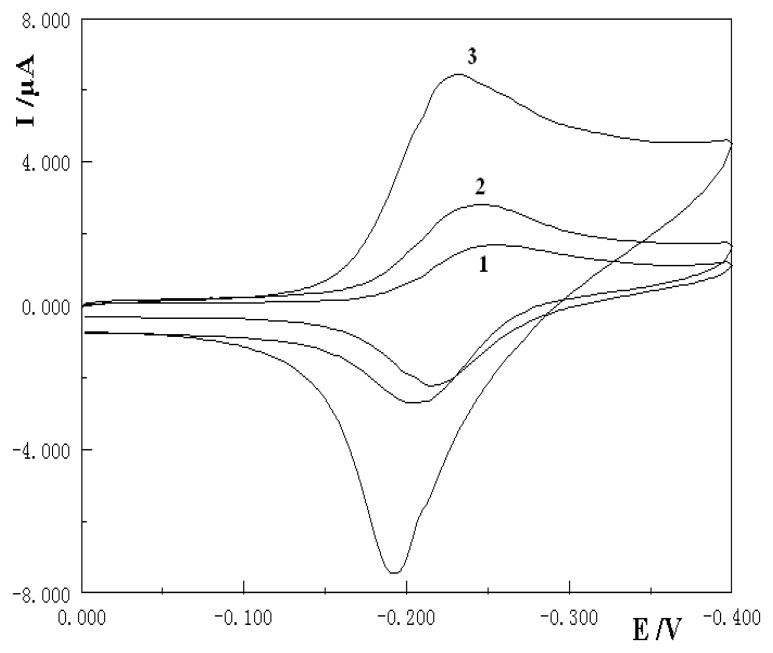
Cyclic voltammograms for the detection of PCR samples by using 1.5×10^-4^ mol/L MB at bare gold electrode (1), NOS ssDNA/MAA/Au electrode (2) and the electrode hybridization with PCR amplified real sample (3).

Then 10 µL of denatured PCR products were dipped on the surface of NOS ssDNA/MAA/Au electrode and the hybridization reaction was taken place at 25 °C for 60 min. After the hybridization reaction the electrochemical detection was applied with the general procedure and the detection results were shown in Figure 6. The MB signal at the bare gold electrode (curve 1) was lower than that at the primer 1 ssDNA/MAA/Au electrode (curve 2). An obvious increase of the MB signal appeared after the hybridization of the probe with real sample PCR products (curve 3). This significant difference of the MB redox signals on the NOS ssDNA/MAA/Au electrode and the hybridized dsDNA/MAA/Au electrode indicated that this electrochemical DNA biosensor could detect the PCR amplified product effectively.

## 4. Conclusions

In this paper a new genetically modified organism electrochemical DNA biosensor was constructed and applied to the directly detection of PCR product from NOS gene using methylene blue as electrochemical indicator. The single-stranded DNA was immobilized on the mercaptoacetic acid self-assembled monolayer electrode to form an ssDNA/MAA/Au electrode, which could hybridize with the complementary ssDNA to form a dsDNA/MAA/Au electrode. The electrochemical behaviors of MB on different kinds of DNA modified electrodes were carefully investigated. The coupling of electrochemical detection with PCR amplification showed the advantages of higher sensitivity of detection with low-cost equipment.

## Figures and Tables

**Scheme 1.**
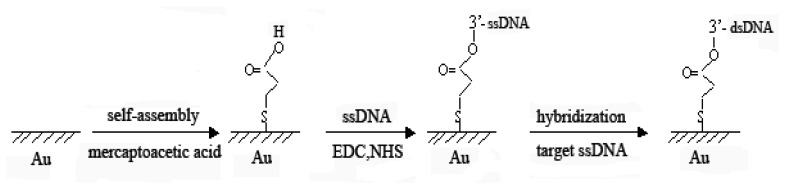
Schedule for the preparation of self-assembled monolayer DNA modified electrode.

**Table 1.** Electrochemical data of MB interaction with ssDNA and dsDNA in solution.

Solution	E_pa_/V	E_pc_/V	i_pa_/µA	i_pc_/µA	ΔE_p_/mV	E^0^′/V
MB	‐0.170	‐0.208	‐2.904	2.971	38	‐0.189
MB+ssDNA	‐0.178	‐0.212	‐1.661	2.301	34	‐0.195
MB+dsDNA	‐0.186	‐0.210	‐0.890	1.626	24	‐0.198
